# In Vivo Nanodiamond Quantum Sensing of Free Radicals in *Caenorhabditis elegans* Models

**DOI:** 10.1002/advs.202412300

**Published:** 2025-01-15

**Authors:** Siyu Fan, Yue Zhang, Anna P. Ainslie, Renée Seinstra, Tao Zhang, Ellen Nollen, Romana Schirhagl

**Affiliations:** ^1^ Department of Biomaterials & Biomedical Technology (BBT) University Medical Centre Groningen (UMCG) Antonius Deusinglaan 1 Groningen 9713 AV The Netherlands; ^2^ European Research Institute for the Biology of Ageing (ERIBA) University Medical Centre Groningen (UMCG) Antonius Deusinglaan 1 Groningen 9713 AV The Netherlands

**Keywords:** *C. elegans*, diamonds, nanodiamonds, NV centers, quantum sensing, relaxometry

## Abstract

Free radicals are believed to play a secondary role in the cell death cascade associated with various diseases. In Huntington's disease (HD), the aggregation of polyglutamine (PolyQ) not only contributes to the disease but also elevates free radical levels. However, measuring free radicals is difficult due to their short lifespan and limited diffusion range. Here, a quantum sensing technique (T1 relaxometry) is used that involves fluorescent nanodiamonds (FND). Nitrogen vacancy (NV) centers within these nanodiamonds change their optical properties in response to magnetic noise, which allows detecting the unpaired electron from free radicals. This method is used to monitor the production of free radicals inside Caenorhabditis elegans models of Huntington's disease in vivo and in real‐time. To investigate if radical generation occurs near polyglutamine expansions, a strain expressing Q40 yellow fluorescent protein (Q40::YFP, polyglutamine expansion overexpressed in the muscle) is used. By applying T1 relaxometry on FNDs in the body wall muscle, it is found that the production of free radicals significantly increase when PolyQ is expressed there (compared to the FNDs in intestine). The technique demonstrates the submicrometer localization of free radical information in living animals and direct measurement of their level, which may reveal the relation between oxidative stress and Huntington's disease.

## Graphical abstract

The aim of this article is to demonstrate diamond‐based quantum sensing in vivo. More specifically, nitrogen vacancy centers in diamond are used for measuring free radical generation in a Huntington disease model in *C. elegans*. Quantum sensing allows localized measurements in the muscles and in the intestine. It is also possible to follow free radical generation while inducing polyQ formation.

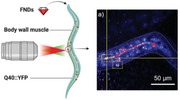

## 1 Introduction

Huntington's disease (HD) is a genetic neurodegenerative disorder marked by abnormal movements, psychiatric symptoms, and cognitive decline.^[^ ^1^ ^]^ Its prevalence ranges from 0.4 to 7.7 per 100 000 people, higher in Western countries. HD is caused by a mutation on chromosome 4, leading to toxic huntingtin protein aggregates when CAG repeats exceed 35–40.^[^ ^2^, ^3^ ^]^ Protein aggregates represent a pathological feature of polyQ‐mediated diseases, and their formation has been widely observed in various experimental setups, suggesting a link between polyQ aggregation and toxicity.^[^ ^2^, ^4^, ^5^ ^]^ While these aggregates are well‐known, their exact role in neural degeneration remains unclear.^[^ ^6^ ^]^

Free radical generation plays a pivotal role in the progression of HD, although it remains uncertain whether they are causative or a consequence of other pathological events.^[^ ^7^, ^8^, ^9^ ^]^ Moreover, antioxidant therapies have not yielded the expected outcomes, highlighting the need for a better understanding of free radical involvement in HD.^[^ ^10^, ^11^, ^12^ ^]^ However, precise intracellular origins of free radicals pose a significant challenge since they are short‐lived and reactive.^[^ ^13^ ^]^

Currently, various methods exist for measuring free radical generation, including indirect approaches and imaging‐based techniques.^[^ ^14^ ^]^ Indirect methods measure reaction products with radicals or specific molecules produced in response to radical generation. However, these methods are usually destructive and do not offer spatial resolution.^[^ ^15^, ^16^ ^]^ Imaging‐based techniques include optical labeling with microscopic tools^[^ ^17^ ^]^ or spin labeling with magnetic resonance techniques like magnetic resonance imaging (MRI) or electron spin resonance (ESR). However, these methods typically measure the cumulative history of reaction products (all reaction products that have been generated between adding the compound and the measurement) rather than the current state. Since reaction molecules can freely diffuse during this time, the spatial resolution is limited. Additionally, these molecules can react with radicals, thereby affecting the radical concentration, and they often possess some degree of inherent toxicity. Lastly, many of these probes exhibit cross‐reactivity with other reactive molecules or specific enzymes.

Quantum sensing, which is based on NV centers in diamonds offers an attractive alternative.^[^ ^18^, ^19^ ^]^ These NV centers emit unprecedentedly stable red fluorescent that changes in intensity based on their spin state.^[^ ^20^, ^21^, ^22^, ^23^ ^]^ As a result, one can use this fluorescence to measure magnetic fields or magnetic noise.^[^ ^24^, ^25^, ^26^ ^]^ Recently this technique has been utilized to measure free radical generation in living cells.^[^ ^27^ ^]^ There the technology has been used for instance to measure stress responses in cancer cells,^[^ ^28^ ^]^ bacteria,^[^ ^29^ ^]^ immune cells,^[^ ^30^, ^31^ ^]^ yeast cells^[^ ^32^ ^]^ or as a response of cells toward viruses.^[^ ^33^ ^]^ Recently, we have also demonstrated that quantum sensing can reveal the stress response at the location of polyQ in cells.^[^ ^34^ ^]^ Here we demonstrate the first in vivo quantum sensing of free radicals in the nematode *C. elegans* where nanodiamonds have so far only been used for fluorescent labeling^[^ ^35^, ^36^, ^37^ ^]^or temperature sensing.^[^ ^38^ ^]^ *C. elegans* is a nematode and a common model organism in biology. It is widely used since it is easy and fast to breed and there are numerous disease models available in *C. elegans*.

To investigate free radical measurements using T1 relaxometry, we initially employed the worm model GA480, which lacks the double superoxide dismutase genes sod‐2 and sod‐3 (mitochondrial sod genes), to serve as a positive control.^[^ ^39^ ^]^ Superoxide dismutase (SOD) is an antioxidant enzyme responsible for converting the reactive oxygen species superoxide into hydrogen peroxide, which is then further converted into water. Thus, the double mutant of sod genes will lead to an increase in the oxidative stress level.^[^ ^40^ ^]^

For studying Huntington's disease in *C. elegans* models, the introduction of disease‐length polyglutamine tracts into various cell types has been demonstrated before.^[^ ^3^, ^41^, ^42^ ^]^ In this study, our primary experimental model involves utilizing a worm model of HD, where 40 glutamines are linked to yellow fluorescent protein (YFP) in body wall muscle cells (AM141 Q40::YFP). This model allows us to investigate the phenotypic effects resulting from polyglutamine toxicity and assess the levels of oxidative stress in various body parts. As a control, a model with only YFP was used (OW450 Q0::YFP).

**Figure 1:**
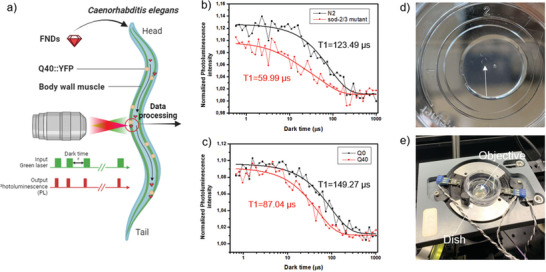
Experiments conducted in this article. a) Schematic representation of the diamond‐based quantum sensing in this article. Nanodiamonds are ingested by *C. elegans* and taken up by cells. Quantum sensing experiments were conducted near the surface where nanodiamond colocalize with Q40::YFP. Representative relaxation curves from nanodiamonds in b) a day 1 adult wild type N2 worm and in a sod‐2/3 mutated worm; c) a control Q0 and a polyglutamine (PolyQ)‐stressed Q40 worm. The NV centers are brought into the bright ms = 0 state. Then we checked after different dark times if the NV centers have remained in this state or returned to the darker equilibrium between ms = 0 and ms = ±1. This process is accelerated by free radicals. d) *C. elegans* are immobilized in agar pads on a glass bottom Petri dish for measurement. e) The dish was placed on the setup.

Here we demonstrate the first diamond‐based free radicals quantum sensing experiments in vivo. **Figure** **1** shows a summary of the experiments conducted in this article. We were able to show the elevated free radical levels in SOD mutant worms and Q40‐expressing worms, with differential levels observed in different body parts of Q40 worms, particularly those closer to polyQ aggregates, indicating oxidative stress induced by polyQ toxicity.

## 2 Results and Discussion

### 2.1 Expression of PolyQ, Motility, and ATP Test of Worms

**Figure 2:**
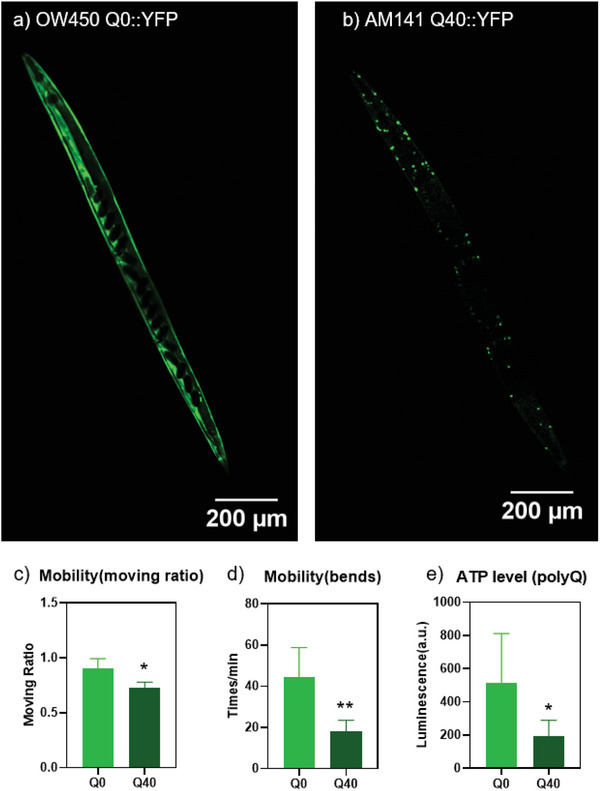
Expression of polyQ expansions in day 1 adult *C. elegans*. a) Q0, b) Q40. Expression of polyQ expansions in *C. elegans* muscle results in a motility defect as indicated in c) the ratio of moving worms among all worms, d) the average bending times of each worm per minutes. e) ATP measurement of day 1 adult Q0 and polyglutamine (polyQ)‐ stressed Q40 worms. Error bars denote standard deviations of three replications. Statistical significance, evaluated using t‐test, is shown as * for *p* ≤ 0.05, ** for *p* ≤ 0.01.

The aggregation and toxicity of polyQ proteins with different length have been studied before in young adult worms (day 3–4).^[^ ^3^ ^]^ As a result, Q40 was found to be the threshold to induce toxicity. Here, we examined day 1 adult worms expressing Q0 and Q40 (labeled by YFP). In Q0 adult animals, we observed diffuse fluorescence distribution in expressing cells (**Figure** **2**a). In contrast, animals expressing Q40 (Figure 2b) exhibited dotted fluorescence near body muscle indicating that polyQ proteins had aggregated.^[^ ^3^, ^43^ ^]^

To assess the motility, the paralysis rate and bending times of 100 worms from different groups were analyzed. The motility difference (Figure 2c,d) in day 1 between Q0 and Q40 adult worms indicated the defects caused by polyQ aggregates. A significant decline in moving ratio can be seen between Q0 and Q40 worms. Among the moving worms, the Q40 group exhibited less bending times compared to Q0 worms. These findings are aligned with previous results.^[^ ^3^, ^41^ ^]^ The motility defect was also linked to cellular toxicity, which was evaluated by measuring ATP levels in worms. As shown in Figure 2e, Q40 worms exhibited a notable reduction in ATP levels compared to the Q0 group.

### 2.2 FNDs Uptake and Distribution

To prevent the accumulation of FNDs and to facilitate the particle uptake by worms, we coated the particles with bovine serum albumin (BSA) as recommended.^[^ ^37^ ^]^ In Figure S1 (Supporting Information), the average hydrodynamic diameter of FND/BSA (220 nm) increased compared to bare FNDs (160 nm), indicating that BSA can be physically absorbed on the surface of FNDs and that there is some aggregation. BSA coating of nanodiamonds has been shown before and only slightly decreases the sensitivity of nanodiamonds to magnetic noise.^[^ ^44^ ^]^ This is likely the case because we select particles with a specific count rate for measurements and thus exclude large aggregates. It is also worth noting that radicals are relatively small and can probably still get close to the surface despite the coating.

**Figure 3:**
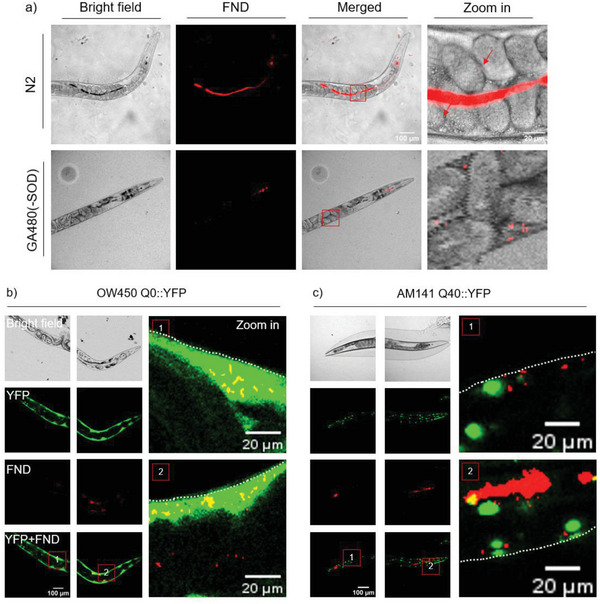
The distribution of FND/BSA particles in a) N2 wild type and GA480 (sod2/3 double mutants) worms. b) OW450 Q0::YFP and c) AM141 Q40::YFP worms. The border of worms is indicated by dashed line in zoom in pictures. The zoom in pictures showed the area highlighted in red boxes in left panel.

FND/BSA were orally administered into worms. **Figure** **3**a displays a fluorescence and bright field image of typical N2 wild‐type and GA480 (sod‐2/sod‐3 double mutant) of day1 adult worms. Similarly, as found before,^[^ ^37^, ^45^ ^]^ bright red fluorescence from FNDs was easily visible in the lumen of the digestive system. Some of the particles were also found in adjacent cells, which indicates that FND/BSA can cross the intestinal cells and diffuse to other parts in worms. Instead of being excreted, those particles that crossed the intestine can be kept for longer time in worms, thus facilitating further measurement. The same particle incubation process was applied to OW450 Q0::YFP and AM141 Q40::YFP worms. Figure 3b,c shows the distributions of FND/BSA in two worms. Besides the FND/BSA particles distributed within the digestive lumen, further colocalization suggests that some particles diffused to the body wall muscle area and colocalized with diffused YFP (Figure 3b) or stayed near the polyQ aggregates (Figure 3c). FNDs have also been found in similar locations earlier in other worms.^[^ ^37^, ^45^ ^]^

### 2.3 FND Biocompatibility

**Figure 4:**
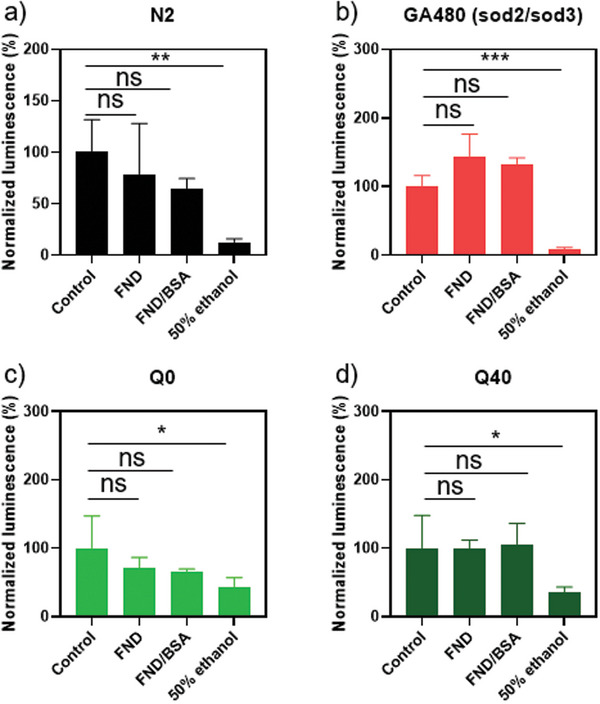
ATP levels were assessed through a Cell Titer assay, repeated three times for diverse worms a) N2 wild type; b) GA480 (sod2/3 double mutants) worms; c) OW450 Q0::YFP; and d) AM141 Q40::YFP worms after incubating with FND, FND/BSA or 50% ethanol (positive control). In each panels, the data was normalized by defining the mean value of control group as 100% and set 0 as 0%. Error bars denoted standard deviations. Statistical significance, evaluated using one‐way ANOVA, is shown as ns (not significant) *p* > 0.05, * for *p* ≤ 0.05, ** for *p* ≤ 0.01, *** for *p* ≤ 0.001.

To evaluate the biocompatibility of nanodiamonds, we conducted a CellTiter assay (**Figure** **4**). In this assay, the Adenosine triphosphate (ATP) content of the sample is measured and normalized by setting the mean luminescence value of control group as 100%. On day1 adult worms exposed to 50 µg mL^−1^ of bare FNDs or BSA‐coated FNDs for 5 h. To serve as a positive control, 50% ethanol was used. Importantly, we observed no notable difference in cell viability between the control and worms exposed to different FND types, indicating the biocompatibility of FNDs with *C. elegans*. In Figure 4c, the P values between FND and 50% ethanol, as well as between FND/BSA and 50% ethanol, were 0.06 and 0.08, respectively. While these values are slightly above the threshold for statistical significance, they suggest only a minor potential influence of the particles on the worms. These results align with previous literature reporting the favorable biocompatibility of FNDs in *C. elegans*.^[^ ^37^ ^]^ This finding is also in line with the biocompatibility that was observed in different cell types as well as in animal models.^[^ ^38^, ^46^, ^47^ ^]^

### 2.4 Reactive Oxygen Species (ROS) Detection by Dihydroethidium Assay

**Figure 5:**
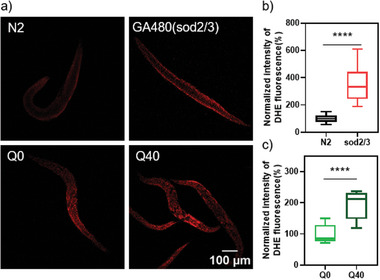
The level of ROS in different day 1 adult *C. elegans* strains as measured by a DHE assay. a) Confocal images of DHE channel. An increase in red fluorescence in mutated worms can be seen. The average DHE fluorescence intensity of day 1 adult nematodes from panel (a) was quantified by FIJI. b) The comparison between N2 and sod2/3 mutated worms. c) The comparison between Q0 and Q40 worms. Whiskers represent the lowest and highest data points. The line splitting the box represents the median value, the upper and bottom box edges represent the higher and lower quartile, respectively. Ten worms were analyzed each time and three independent experiments were performed. Data between each group were normalized by defining the mean value of control group as 100% and set 0 as 0%. Then analyzed by an unpaired t test. **** for *p* ≤ 0.0001.

To investigate whether different mutants experience increased oxidative stress, we measured ROS levels in nematodes treated with dihydroethdium (DHE). DHE is known to be easily oxidized by various oxidants like superxoxide, producing 2‐hydroxyethdium, then binding with DNA and emit red fluorescence.^[^ ^48^ ^]^ To separate the signal of YFP and 2‐hydroxyethdium, the emission wavelength was set as 610–650 nm. In **Figure** **5**b, the quantification of the red signal in N2 and GA480 (sod2/3) indicated an increased oxidative stress raised in sod2/3 double mutants, consistent with the function of sod‐2 and sod‐3 as mitochondrial antioxidant enzymes, converting superoxide to hydrogen peroxide, and subsequently to water.^[^ ^41^ ^]^ A significant difference of ROS levels was also found between Q0 and Q40 worms, suggesting that polyQ proteins also increase oxidative stress in worms (Figure 5c). The results are consistent with previous observation, showing that antioxidant enzymes, like sod‐3, are upregulated in Q40 worms.^[^ ^41^ ^]^ Notably, YFP itself also influenced oxidative stress levels in *C. elegans*, similar to what has been reported before for cells^[^ ^49^, ^50^ ^]^ and mice.^[^ ^51^ ^]^ In *C. elegans*, we indeed saw the difference in Figure S2b (Supporting Information).

### 2.5 Free Radical Detection by T1 Relaxometry

Although DHE fluorescence is frequently utilized to assess reactive oxygen species (ROS) levels, it provides an indirect measure of 2‐hydroxyethdium rather than directly detecting superoxide. Additionally, challenges such as limited photostability and spatial resolution impede long‐term tracking at precise locations.

**Figure 6:**
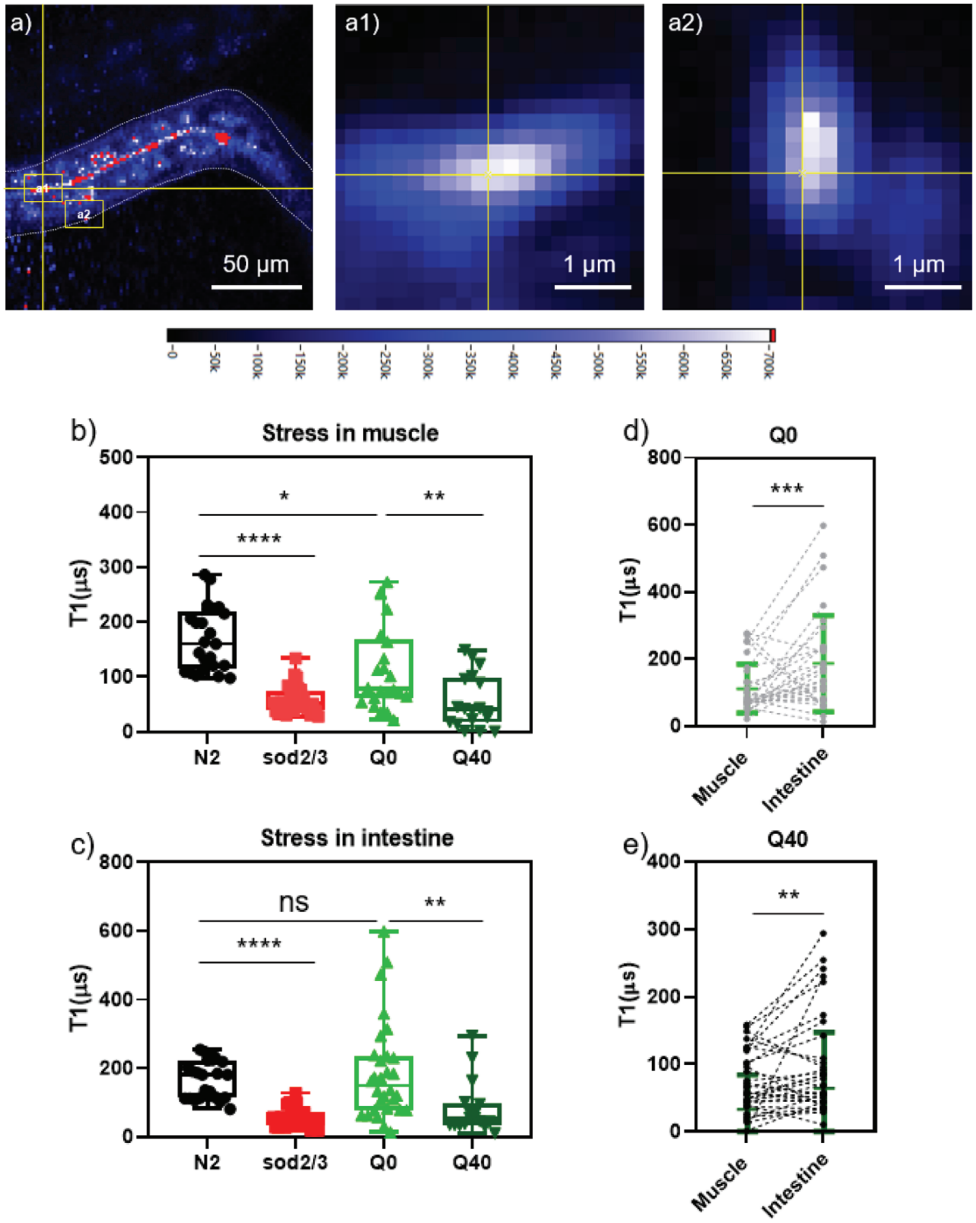
Free radical detection by T1 relaxometry in different worm strains, data was obtained from multiple independent experiments, ≈30 measurements were performed for each group. a) Representative fluorescence image of FND/BSA in a Q40 worm. The white dashed line is the body border. The intensity bar shown on the bottom is shown in photon counts/s. a1) Zoom in of an intestinal particle from a) (at the location of the yellow cross point). Photon counts are 1.2 × 10^7^ counts s^−1^. a2) a zoom in on a particle from panel a) near the body muscle wall with 1×10^7^ counts s^−1^. T1 relaxometry measurements in FND/BSA particles b) near body muscle or c) in intestine are shown from N2, sod2/3 double mutant worms, Q0 and Q40 worms. To differentiate the free radical levels in different parts of worms, FND/BSA particles from intestine or body muscle wall in the same worm were measured. Dashed lines show individual measurements in a single d) Q0 worm; e) Q40 worm. In (b) and (c), Whiskers represent the lowest and highest data points, the line splitting the box represents the median value. The standard deviations are listed in Table S2 (Supporting Information). The upper and lower box edges represent the higher and lower quartile, respectively. Significance between groups was analyzed by an unpaired t test. * for *p* ≤ 0.05, **** for *p* ≤ 0.0001. In (d) and (e), error bars represent standard deviations of three independent experiments with 30 worms measured. Significance between groups was analyzed by a paired t test. ns = no significant difference, * for *p* ≤ 0.05, ** for *p* ≤ 0.01, *** for *p* ≤ 0.001, and **** for *p* ≤ 0.0001.

In contrast, T1 relaxometry, which relies on sensing FND surrounding magnetic noise, offers the capability to detect radical responses near polyglutamine aggregates in the body wall muscle of living animals. Here it is worth mentioning that the worms and thus the nanodiamonds move during the measurement. Typically, we observed a movement of 0–1 µm during our measurement. If the nanodiamonds are in the same location as the polyglutamine aggregates (for instance if they are in the same vesicle or if the entire worm moves), they are expected to move together and their distance remains constant. Also free radicals can diffuse through the cell but their diffusion range is limited by their lifetime. They have to be within a few tens of nm to be detectable by nanodiamonds. The experimental setup, as previously described,^[^ ^32^ ^]^ utilized a green pulse sequence (Figure 1a) to perform relaxometry. In a single measurement, a nanodiamond with photon counts ≈10^7^ was selected. The autofluorescence photon counts from worms were below 10^6^, so easily to be distinguished from the FNDs. A FND was located at desired position within worms was first selected (**Figure** **6**a), then tracked during laser pulsing (Figure 6a1,a2), and subjected to biexponential decay analysis. A 650 nm filter was applied to remove YFP signal. The T1 value, quantified from the decay velocity of biexponential curves, reflects the magnetic noise in the surroundings.^[^ ^52^ ^]^ Lower T1 values indicate higher radical concentration. As the 120 nm FND contains ≈900 NV centers, the data distribution of T1 values is relatively narrow, which makes the results more reproducilble (Figure S3a, Supporting Information). Also, when compared T1 values in MillQ water and in N2 worms, a significant difference with * for *p* ≤ 0.05 was found, indicating that despite the inherent distribution of T1 values, the method is able to detect environmental changes.

As shown in Figure 1b, in a single FND measurement near body muscle, a lower T1 value was observed in sod‐2/3 mutated worms compared to wild type N2 worms, indicating the applicability of T1 measurement in living animals and its correlation with higher radical concentration. We then continued measurement on the particle in polyQ‐stressed Q40 worm, where a faster decay can be seen compared to the Q0 worm. That it is possible to observe differences between Q0 and Q40 from single FND measurements, indicates the high sensitivity of T1 relaxometry.

The measurement was further repeated in over 30 worms for each group. When comparing N2 and SOD mutated worms, significance with **** for *p* ≤ 0.0001 was observed in both body muscle and intestine part, indicating the stress from SOD mutant existed among the entire body of worms.

In contrast, the comparison between N2 and Q0 worms revealed a significant difference only in the body muscle (Figure 6b) but not in the intestine (Figure 6c). Since Q0::YFP is predominantly expressed in the body muscle (Figures 2 and 4), this result suggests that oxidative stress is induced by YFP expression. This finding aligns with Figure S2a (Supporting Information), which shows that the presence of foreign YFP protein alone is sufficient to trigger oxidative stress.

In Q40 worms’ body muscle (Figure 6b), where a significant decrease (**p ≤ 0.01) in T1 was observed compared to its control Q0 worms (Figure 6b), indicating the contribution of Q40 to elevated free radical level. We observe a larger spread in the data for Q0 than for Q40. This difference can probably be attributed to the nonlinear concentration dependence of T1 that is more sensitive to changes at lower concentrations of T1. In intestine (Figure 6c), a significant difference was also observed between Q0 and Q40 worms. As various stress responses, including SKN‐1‐mediated stress response,^[^ ^53^ ^]^ DAF‐16‐mediated oxidative stress response,^[^ ^54^ ^]^ and hypoxia response,^[^ ^55^ ^]^ were activated in Q40 worms, which might give the intestinal stress.

The change in free radical concentrations between Q0 and Q40 worms differs across tissues, as shown by the T1 calibration curve (Figure S4, Supporting Information). In the intestine, the magnetic signal concentration increased from ≈0.3 nm in Q0 to 10.4 nm in Q40 worms. However, in the body muscle, the increase was far greater, from 2.9 nm in Q0 to 9100 nm in Q40, indicating that polyQ aggregates in body muscle generate significantly more free radicals than those in the intestine.

To further elucidate the location of free radical production within worms, T1 measurements were performed in different parts of the worms. In AM141 Q40::YFP adult worms, where Q40 primarily accumulated in the body wall muscle cells, a significant increase in free radical levels was observed compared to the intestine (Figure 6e, **p ≤ 0.01). This suggests the oxidative stress generated by polyQ aggregates and YFP. Similarly, a significant increase in free radical levels was observed in Q0 worms' body muscle wall (Figure 6d, ***p ≤ 0.001) compared to the intestine, further indicating that YFP expression directly contributes to free radical generation.

## 3 Conclusion

This study demonstrates the feasibility of using T1 relaxometry for measurement of free radical levels in living organisms, specifically in a *C. elegans* model of Huntington's disease (HD). We observed a significant decrease in T1 values in worms expressing disease‐length polyglutamine tracts, indicating elevated levels of oxidative stress. Furthermore, by performing T1 measurements in different body parts, we were able to differentiate the contribution of polyQ aggregates and exogenous YFP proteins to free radical generation. These findings underscore the potential of T1 relaxometry as a sensitive tool for investigating oxidative stress dynamics in disease models. Moreover, our study highlights the utility of quantum sensing techniques, such as T1 relaxometry, for probing biological processes at the nanoscale level in vivo, paving the way for further insights into the pathogenesis of neurodegenerative disorders and the development of targeted therapeutic strategies.

## Conflict of Interest

RS is founder of the spin off company, QTsense which commercializes quantum sensing equipment. The other authors have no conflict of interest to disclose.

## Supporting information



Supporting Information

## Data Availability

The data that support the findings of this study are available from the corresponding author upon reasonable request.
