# Supplementary material for: In Vivo Nanodiamond Quantum Sensing of Free Radicals in Caenorhabditis elegans Models
Source: Adv Sci (Weinh). 2025 Jan 15;12(14):2412300. doi: 10.1002/advs.202412300 (PMC11984841; doi:10.1002/advs.202412300)
Supplement: Supplementary file 1 — Supporting Information [file ADVS-12-2412300-s001.docx]

**Supplementary Material**

**Materials and Methods**

**FND preparation**

The FND particles (Adamas Nanotechnologies, North Carolina, USA) possess a hydrodynamic diameter of 120 nm. Synthesized through high-pressure high-temperature techniques, followed by high-temperature annealing^1^, they undergo a cleaning process in oxidizing acid, resulting in surface oxygen termination^2^. Each measurement in this study represents an average of all nitrogen-vacancy (NV) centers within a particle, enhancing the reliability of the signals emitted by the particles. These biocompatible FNDs maintain stable fluorescence post cellular uptake, rendering them suitable for various biological applications^3,4^.

Bovine Serum Albumin (BSA) coating was conducted by dispersing the ultrasonicated FNDs (1 mg/mL) in 0.6 mg/mL BSA solution (Merck KGaA, Darmstadt, Germany) at a ratio 1:1. The conjugation proceeded for 5min at room temperature, resulting in the formation of BSA-coated FND. The average sizes of the Bare FNDs and BSA-coated FND particles were assessed using the Malvern ZetaSizer Nanosystem (Malvern Instruments Ltd., UK).

**C. elegans strains and maintenance**

The strain AM141 rmIs133 [P(unc-54)Q40::YFP]X (polyglutamine expansion overexpressed in muscle and C-terminally fused to YFP) was used. OW450 Q0::YFP (expressing YFP only), N2 wild type and GA480 [sod-2(gk257) I; sod-3(tm760) X] (sod-2/sod-3 double mutants) strains were used as controls. Strains were maintained at 20°C on nematode growth media (NGM) plates seeded with Escherichia coli OP50 according to the standard protocol^5^.

**Feeding**

Approximately 30−50 adults of growing hermaphrodites were picked into 500 ul M9 buffer (5 mM potassium phosphate, 1 mM CaCl_2_, and 1 mM MgSO_4_), then centrifuging at 3000 rpm for 5min to wash out the adhered bacteria. After washing 3 times, worms were transferred to 500 ul FND or FND/BSA solution (50 ug/mL), incubated for 5 h at 20 °C, with shaking in between to avoid the sink of FNDs.

**Imaging**

After 5h incubation with FNDs, adult worms were wash by M9 buffer for 3 times to clean the FNDs adhered on the skin. Then, worms were picked onto an 35mm glass bottom Petri Dish. Afterward an agar pad was put on the top to sandwich the worms. Z-stack confocal images were taken with a Leica SP8x microscope (Leica, Germany). FNDs were detected at 561/659 nm, and YFP was imaged at 513/530 nm. For FND location experiment, 10 day 1 adult worms were imaged each time and this was repeated for 3 times.

**ATP level evaluation in C.elegans**

Following the optimized protocol^6^, the CellTiter-Glo luminescent assay was conducted to assess metabolic activity (ATP content) in worms exposed to a specific compound. The assays were performed in 96-well plates, each containing 30 worms suspended in 50 μl of M9 buffer. The experiments were conducted using 1-day-old adult worms. C. elegans were incubated in media for 30 minutes at room temperature, with wells containing media alone serving as background controls. Subsequently, 50 μl of CellTiter-Glo solution was added to each well, and the plates were incubated on a shaker for 15 minutes at room temperature, followed by a 10-minute resting period. Luminescence intensities, indicative of ATP content, were then measured using the FLUOstar Omega Microplate Reader (BMG Labtech, De Meern, The Netherlands), with 50% ethanol included as a positive control.

**Dihydroethidium (DHE) assay**

As suggested previously^7^, we evaluated reactive oxygen species (ROS) levels utilizing dihydroethidium (DHE), a ROS-responsive dye that emits red fluorescence upon oxidation. 10 worms were exposed to 3 μM DHE solution in DMSO, incubated for 1 hour with agitation. Following incubation, worms underwent three washes in M9 buffer before immobilization for imaging. To filter the signal of YFP, emission wavelength of 610-650nm was applied. The experiments were repeated for 3 times.

To quantify the fluorescence intensity, FIJI (http://rsbweb.nih.gov/ij/download.html) was used. Firstly, select the worms by ROI tool. Then, measure the *area integrated intensity* and *mean grey value* from *Set Measurements* menu. Repeating the steps for 10 worms each group each time, recording the data to calculate the corrected total fluorescence (CTF).

CTF=Intergrated Density-(Area selected x Mean fluorescence of background). The corrected data was then normalized by setting the mean value of control group as 100% and 0 as 0% and analysed by Prism.

**Motility Assays**

Following the standard protocol^8^, after washing worms with M9 buffer for 3 times, around 100 animals were transferred to new tracker plates without food, and their movements were recorded with the Wide field tracking platform (WF-NTP) for 30 seconds at a frame rate 20fps. The movies were then analyzed with the WF-NTP software to assess the animals' moving ratio and average bending times. The experiments were repeated for 3 times.

**Free radical measurements in C. elegans by T1 measurement**

A previously described home-built magnetometry setup was employed for T1 measurements^9^. The setup is essentially a confocal microscope with an acousto-optical modulator (Gooch & Housego, model 3350-199) for detection. Glass bottom Petri dishes with adult worms were prepared as decribed in “Imaging” part. To explore in vivo free radical generation in worms near body muscle wall, worms were incubated with 50 µg/mL FND/BSA for 5h, followed by M9 buffer washing. To immobilize worms, 5% agar was used to make a pad (thickness is around 0.2 cm, diameter is around 1cm). The agar pad was attached on a 21cmx21cm cover glass. A 2 ul M9 buffer (contains around 5-10 worms) was dropped on the dish, the glass was then reversely put on the dish to immobilize worms. After sandwiching worms on Petri dish, T1 measurements were performed on FND/BSA particles near body wall. To measure the T1 value in different body parts, within one worm, a particle in intestine and a particle in body muscle were found and measured, the values were linked in the graph. The data in Figure 6 are from multiple independent experiments. The experiments were repeated for 3 times and in total over 30 measurements were done for each group.

In T1 relaxometry measurements, NV defects within diamonds served as the basis for quantum sensing at room temperature. These NV centers in diamonds functioned as detectors of magnetic noise in the surrounding environment. Utilizing the pulse sequence depicted in Figure 1a, relaxometry was conducted, with the decay rate of NV centers correlating with the magnetic noise originating from free radicals.

In a typical T1 measurement, NV centers were initialized into the bright ms=0 state, and observations were made at various time intervals to determine if the NV centers maintained this state or returned to equilibrium between ms=0 and ms=+1 and -1.

During pulsing, the NV centers were stimulated using a series of 5 µs green laser pulses (561 nm), with a dark time (τ) ranging from 200 ns to 1 ms. The resulting signal was filtered at 650 nm. To generate relaxometry curves, the brightness within the initial 0.6 µs of each pulse was plotted against the dark time, as illustrated in Figure 1b. From these curves, T1 values were calculated using a bi-exponential model comprising a short T_S_ and a long T_L_ component, where T_L_ represents the final T1 value.

*
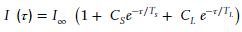
* **Equation 1**

The photoluminescence intensity at long dark times (𝜏), denoted as *I_∞_*, represents the final thermal equilibrium. *C_S_* and *C_L_* refer to the short and long contrast of the relaxation curves, respectively, within the bi-exponential model, as elaborated in prior work^10,11^. This model was adapted from the single exponential model, which is typically used for single NV centers since the single exponential was insufficient to fit the data for the more complex ensemble. We used the longer constant since this constant better predicted known concentrations of gadolinium. The time required to reach the equilibrium condition shortened in the presence of free radicals. The pulsing sequence was repeated 10,000 times for each measurement to ensure a satisfactory signal-to-noise ratio. This resulted in a total measurement time of 1 min per T1 measurement. The laser power at the sample location, measured during continuous illumination, was set at 50 µW. This laser power was chosen to minimize cell damage while being sufficiently high to polarize the NV centers.

**Statistical analysis**

Data analysis was carried out using GraphPad Prism version 8.0. The significance of the results was assessed employing either a T-test, one-way or two-way ANOVA test (Tukey multiple comparisons), depending on the nature of the specific experiment. Significance was determined by comparing the experimental groups to the control group. The defined significance levels were as follows: ns (not significant) for p > 0.05, * for p ≤ 0.05, ** for p ≤ 0.01, *** for p ≤ 0.001, and **** for p ≤ 0.0001


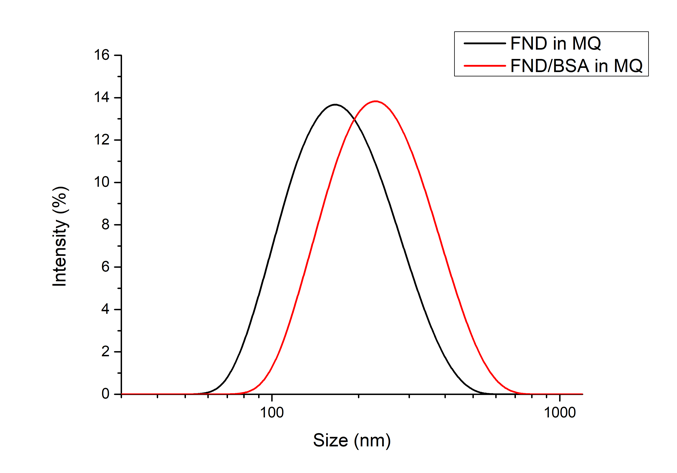

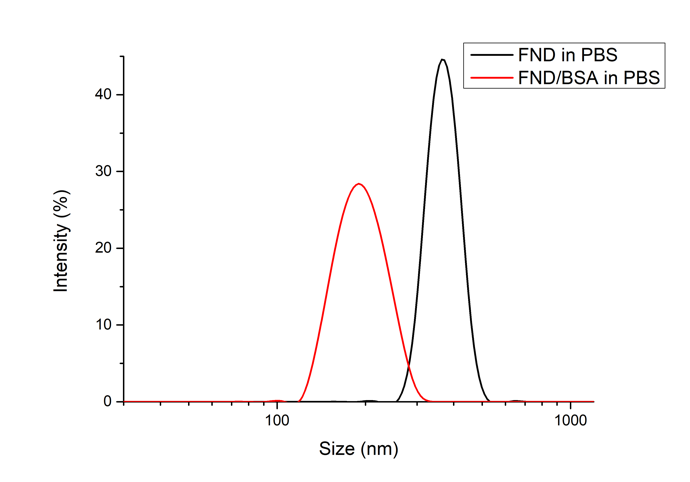


Figure S1. Size measurement of bare FNDs and FND/BSA in MillQ or PBS, measured by the Malvern ZetaSizer Nano system. The size and PDI are indicated in Table S1. The observed increase in particle size suggests that bare FNDs were effectively coated by BSA. Smaller size of FND/BSA in PBS was observed compared to FND, which indicated BSA coating could prevent the aggregation of FND in PBS.

Table S1 Size and polydispersity index (PDI) of FND or FND/BSA.

|  | Size (nm) | PDI |
| --- | --- | --- |
| FND in MillQ water | 160 | 0.12 |
| FND/BSA in MillQ water | 220 | 0.13 |
| FND in PBS | 748 | 0.58 |
| FND/BSA in PBS | 238 | 0.30 |


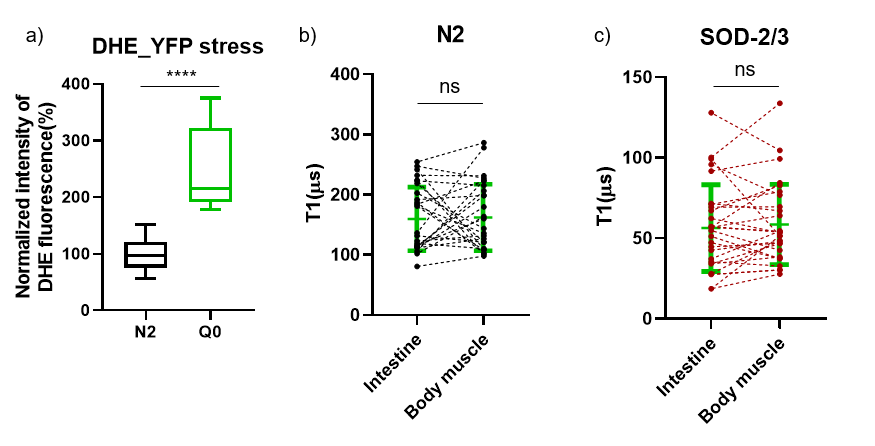


Figure S2 a) The level of ROS in different N2 and Q0 worms, measured by a DHE assay. Whiskers represent the lowest and highest data points, the line splitting the box represents the median value. The upper and lower box edges represent the higher and lower quartile, respectively. Significance between groups was analyzed by an unpaired t test. **** for p ≤ 0.0001. To differentiate the free radical levels in different body parts of worms, FND/BSA particles from intestine or body muscle wall in the same worm were measured. Dash lines show individual measurements in single b) N2 worms; c) sod-2/3 mutated worms. Error bars represent standard deviations of three independent experiments with 30 worms measured. Significance between groups was analyzed by a paired t test. ns=no significant difference.


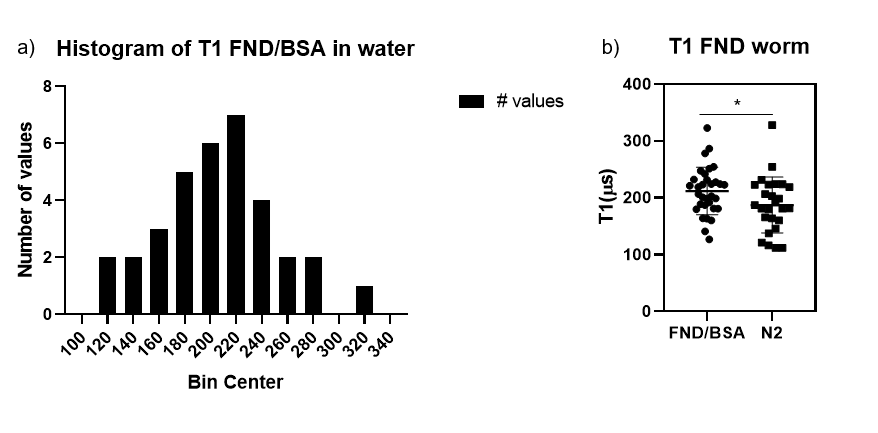


Figure S3, a) Histogram of data distribution of 30 T1 values, measured using FND/BSA particles in MillQ water. b) T1 comparison between FND/BSA particles in water or in N2 worms. * for p ≤ 0.05.


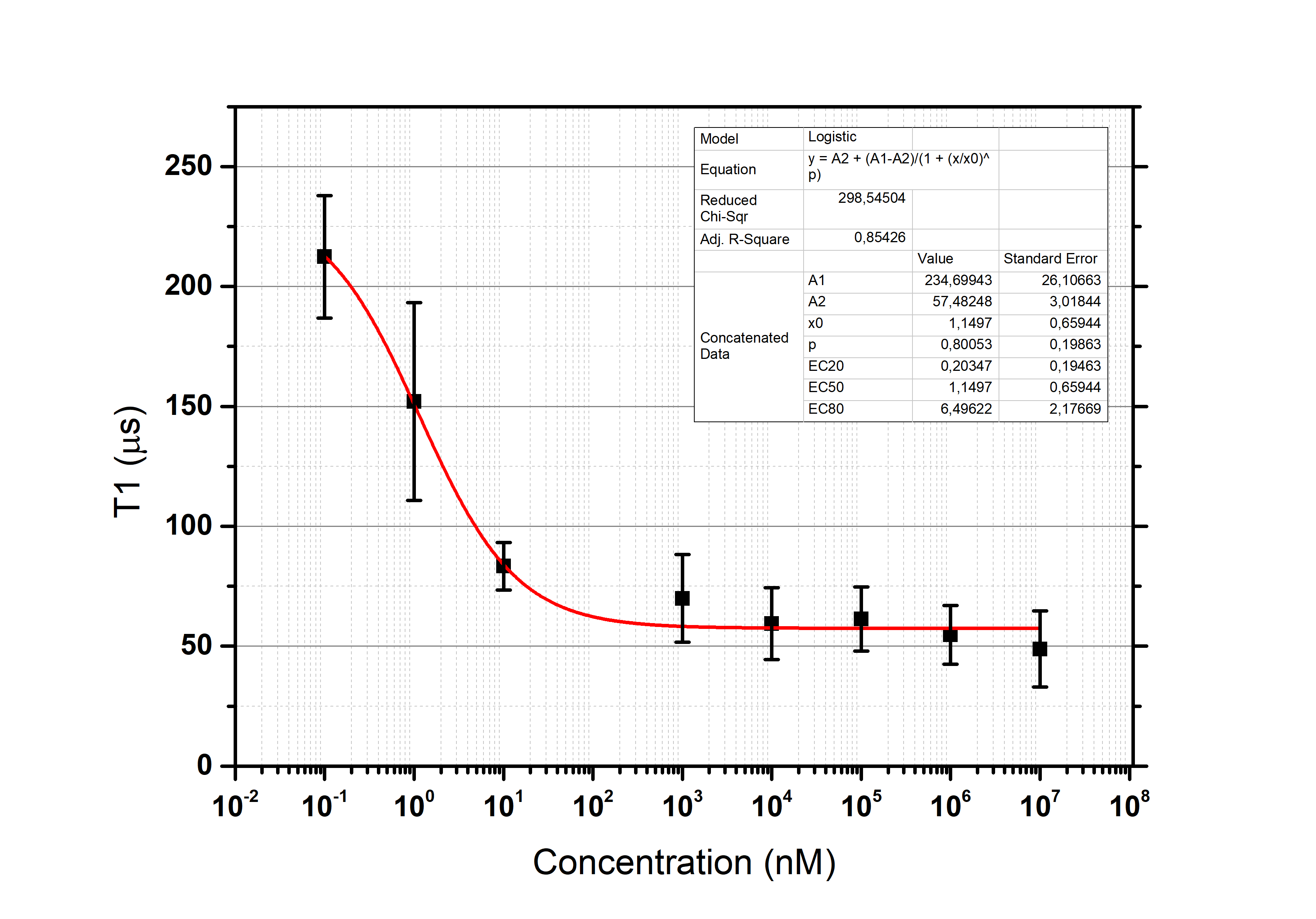


Figure S4, T1 values at difference concentration of Gd^3+^ (serve as stable magnetic signal^10^).

Table S2 The mean values and standard deviations of T1 in Figure 6.

|  | Intestine (µs) | Muscle (µs) |
| --- | --- | --- |
| N2 | 165.5±54.81 | 168.2±57.28 |
| SOD2/3 mutant | 56.65±27.2 | 58.88±25.15 |
| Q0 | 187.4±143.6 | 113.7±77.05 |
| Q40 | 82.37±77.32 | 54.90±48.50 |

Reference

(1) Shenderova, O. A.; Shames, A. I.; Nunn, N. A.; Torelli, M. D.; Vlasov, I.; Zaitsev, A. Review Article: Synthesis, Properties, and Applications of Fluorescent Diamond Particles. *Journal of Vacuum Science and Technology. B, Nanotechnology & Microelectronics* **2019**, *37* (3), 030802. https://doi.org/10.1116/1.5089898.

(2) Schirhagl, R.; Chang, K.; Loretz, M.; Degen, C. L. Nitrogen-Vacancy Centers in Diamond: Nanoscale Sensors for Physics and Biology. **2014**. https://doi.org/10.1146/annurev-physchem-040513-103659.

(3) Mochalin, V. N.; Shenderova, O.; Ho, D.; Gogotsi, Y. The Properties and Applications of Nanodiamonds. **2012**. https://doi.org/10.1038/NNANO.2011.209.

(4) Mohan, N.; Chen, C.-S.; Hsieh, H.-H.; Wu, Y.-C.; Chang, H.-C. In Vivo Imaging and Toxicity Assessments of Fluorescent Nanodiamonds in Caenorhabditis Elegans. **2010**. https://doi.org/10.1021/nl1021909.

(5) Brenner, S. THE GENETICS OF CAENORHABDZTZS ELEGANS.

(6) *1764 AGING*; 2023. www.aging-us.com.

(7) Machiela, E.; Dues, D. J.; Senchuk, M. M.; Van Raamsdonk, J. M. Oxidative Stress Is Increased in C. Elegans Models of Huntington’s Disease but Does Not Contribute to Polyglutamine Toxicity Phenotypes. **2016**. https://doi.org/10.1016/j.nbd.2016.08.008.

(8) Koopman, M.; Peter, Q.; Seinstra, R. I.; Perni, M.; Vendruscolo, M.; Dobson, C. M.; J Knowles, T. P.; A Nollen, E. A. Assessing Motor-Related Phenotypes of Caenorhabditis Elegans with the Wide Field-of-View Nematode Tracking Platform. https://doi.org/10.1038/s41596-020-0321-9.

(9) Loretz, M.; Pezzagna, S.; Meijer, J.; Degen, C. L. Nanoscale Nuclear Magnetic Resonance with a 1.9-Nm-Deep Nitrogen-Vacancy Sensor. **2014**.

(10) Perona Martínez, F.; Nusantara, A. C.; Chipaux, M.; Padamati, S. K.; Schirhagl, R. Nanodiamond Relaxometry-Based Detection of Free-Radical Species When Produced in Chemical Reactions in Biologically Relevant Conditions. **2023**, *30*, 38. https://doi.org/10.1021/acssensors.0c01037.

(11) Vedelaar, T. A.; Hamoh, T. H.; Martinez, F. P. P.; Chipaux, M.; Schirhagl, R. Optimizing Data Processing for Nanodiamond Based Relaxometry. *Adv Quantum Technol* **2023**. https://doi.org/10.1002/QUTE.202300109.
